# Supplementary material for: Rice (Oryza sativa L.) Grain Size, Shape, and Weight-Related QTLs Identified Using GWAS with Multiple GAPIT Models and High-Density SNP Chip DNA Markers
Source: Plants (Basel). 2023 Nov 30;12(23):4044. doi: 10.3390/plants12234044 (PMC10708019; doi:10.3390/plants12234044)
Supplement: Supplementary file 1 [file plants-12-04044-s001.zip › plants-2644637-supplementary.pdf]

Rice (*Oryza sativa* L.) Grain Size, Shape, and Weight-Related QTLs Identified Using GWAS with Multiple GAPIT Models and High-Density SNP Chip DNA Markers

Nkulu Rolly Kabange [1,*], Gamenyah Daniel Dzorkpe [2], Dong-Soo Park [1], Youngho Kwon [1], Sais-Beul Lee [1], So-Myeong Lee [1], Ju-Won Kang [1], Seong-Gyu Jang [1], Ki-Won Oh [1] and Jong-Hee Lee [1,*]

[1] Department of Southern Area Crop Science, National Institute of Crop Science, Rural Development Administration (RDA), Miryang 50424, Republic of Korea; parkds9709@korea.kr (D.-S.P.); kwon6344@korea.kr (Y.K.); pappler@korea.kr (S.-B.L.); olivetti90@korea.kr (S.-M.L.); kangjw81@korea.kr (J.-W.K.); sgjang0136@korea.kr (S.-G.J.); ohkw1004@korea.kr (K.-W.O.)

[2] Council for Scientific and Industrial Research (CSIR), Crops Research Institute, Kumasi 3785, Ghana; gamenyahdaniel@gmail.com

* Correspondence: rollykabange@korea.kr (N.R.K.); ccriljh@korea.kr (J.-H.L.); Tel.: +82-10-5413-4881 (N.R.K.); +82-53-350-1169 (J.-H.L.); Fax: +82-55-352-3059 (J.-H.L.)

Table S1. Normality test of grain traits (GL, GW, GT, LWR, and TGW)

Observations	Grain traits				
	GL	GW	GT	LWR	TGW
Mean	60.1	36.5	2.01	1.77	21.1
Std. Deviation	2.36	1.66	0.0664	0.123	1.29
Std. Error of Mean	0.192	0.135	0.0054	0.01	0.105
Sum	9077	5509	303	267	3189
Skewness	1.48	-1.15	-1.26	4.21	-0.41
Kurtosis	7.15	5.48	7.48	33.7	1.28
D'Agostino & Pearson normality test					
K2 (95.4% of data falls within two standard deviations of the mean)	65.7	49.7	59.6	174	10
P value	<.001	<.001	<.001	<.001	0.007
Passed normality test (alpha=0.05)?	No	No	No	No	No
P value summary	***	***	***	***	**
Shapiro-Wilk normality test					
W	0.885	0.916	0.909	0.722	0.976
P value	<.001	<.001	<.001	<.001	0.009
Passed normality test (alpha=0.05)?	No	No	No	No	No
P value summary	***	***	***	***	**

GL: grain length, GW: grain width, GT, grain thickness, LWR: grain length-to-width ratio, and TGW: thousand grain weight, ***$p<0.001$, **$p<0.01$.

Table S2. Linked Markers to grain length, width, thickness, LWR, and thousand-grain weight.

Trait/QTL	SNP Chip Marker	Chr	Position (bp)	*P-value*	MAF	nobs	Effect	PVE (%)	Allele
GL	BLINK								
qGL1-1^{BFSG}	AX-95918134	1	3820526	4.27E-15	0.02	143	–3.54	72.5	TTWH1
qGL11-1 ^{BFSG}	AX-274862201	11	16356105	8.58E-24	0.06	143	2.35	31.9	Ilpum
qGL2-1^{BFSG}	AX-115751685	2	35100558	1.62E-10	0.34	143	–0.66	22.9	TTWH1
qGL3-1^{BF}	AX-154437636	3	2253428	1.72E-09	0.20	143	0.86	5.5	Ilpum
qGL2-2^{BF}	AX-154880023	2	35133528	2.88E-07	0.29	143	–0.70	3.1	TTWH1
qGL7-1^{BFSG}	AX-153903748	7	6690089	4.09E-12	0.44	143	–0.79	2.6	TTWH1
qGL1-2^{BF}	AX-279584700	1	41489588	6.18E-09	0.13	143	–1.29	1.7	TTWH1
qGL5^{BFSG}	AX-282746698	5	16169537	2.05E-09	0.19	143	0.72	1.4	Ilpum
	FarmCPU								
qGL1-1^{BFSG}	AX-95918134	1	3820526	2.49E-17	0.02	143	–3.72	65.5	TTWH1
qGL11-1 ^{BFSG}	AX-274862201	11	16356105	6.86E-23	0.06	143	2.26	34.5	Ilpum
qGL11-2^{FSG}	AX-115751092	11	2823622	3.79E-07	0.08	143	0.93	14.0	Ilpum
qGL3-2^{Farm}	AX-154551783	3	8242439	5.58E-08	0.17	143	–1.42	8.0	TTWH1
qGL7-1^{BFSG}	AX-153903748	7	6690089	2.67E-09	0.44	143	–0.73	7.6	TTWH1
qGL2-1^{BFSG}	AX-115751685	2	35100558	1.36E-07	0.34	143	–0.56	5.1	TTWH1
qGL5-1^{BFSG}	AX-282746698	5	16169537	1.27E-08	0.19	143	0.67	4.3	Ilpum
qGL3-1^{BF}	AX-154437636	3	2253428	3.00E-09	0.20	143	0.76	3.8	Ilpum
qGL2-2^{BF}	AX-154880023	2	35133528	1.23E-06	0.29	143	–0.64	3.2	TTWH1
qGL1-2^{BF}	AX-279584700	1	41489588	2.95E-08	0.13	143	–1.13	1.3	TTWH1
	SUPER								
qGL1-1^{BFSG}	AX-95918134	1	3820526	4.27E-15	0.02	143	–3.54	72.5	TTWH1
qGL11-1 ^{BFSG}	AX-274862201	11	16356105	6.86E-23	0.06	143	2.26	34.5	Ilpum
qGL11-2^{FSG}	AX-115751092	11	2823622	3.79E-07	0.08	143	0.93	14.0	Ilpum
qGL7-1^{BFSG}	AX-153903748	7	6690089	2.67E-09	0.44	143	–0.73	7.6	TTWH1
qGL2-1^{BFSG}	AX-115751685	2	35100558	1.36E-07	0.34	143	–0.56	5.1	TTWH1
qGL5-1^{BFSG}	AX-282746698	5	16169537	1.27E-08	0.18	143	0.67	4.3	Ilpum
	GLM								
qGL1-1^{BFSG}	AX-95918134	1	3820526	4.27E-15	0.02	143	–3.54	72.5	TTWH1
qGL11-1 ^{BFSG}	AX-274862201	11	16356105	6.86E-23	0.06	143	2.26	34.5	Ilpum
qGL11-2^{FSG}	AX-115751092	11	2823622	3.79E-07	0.08	143	0.93	14.0	Ilpum
qGL7-1^{BFSG}	AX-153903748	7	6690089	2.67E-09	0.44	143	–0.73	7.6	TTWH1
qGL2-1^{BFSG}	AX-115751685	2	35100558	1.36E-07	0.34	143	–0.56	5.1	TTWH1
qGL5-1^{BFSG}	AX-282746698	5	16169537	1.27E-08	0.18	143	0.67	4.3	Ilpum
GW	BLINK								
qGW1-1^{BFSG}	AX-273945773	1	5623288	8.36E-13	0.32	143	0.63	18.9	Ilpum
qGW2-1^{Blink}	AX-279699609	2	10805604	8.17E-18	0.10	143	–1.16	14.9	TTWH1
qGW1-3^{BF}	AX-115791785	1	43103625	9.44E-28	0.30	143	–1.01	8.8	TTWH1
qGW1-4^{Blink}	AX-281116133	1	20864932	1.52E-11	0.17	143	0.56	6.9	Ilpum
qGW6-1^{BF}	AX-115737727	6	28484619	5.27E-07	0.15	143	0.42	6.2	Ilpum
qGW3-1^{Blink}	AX-154073979	3	7895651	9.65E-08	0.33	143	0.46	4.8	Ilpum
qGW3-2^{Blink}	AX-115811160	3	14888685	6.12E-15	0.21	143	0.65	4.1	Ilpum
	FarmCPU								

qGW1-1^{BFSG}	AX-273945773	1	5623288	3.39E-09	0.32	143	0.57		15.5	Ilpum
qGW6-2^{FSG}	AX-273990782	6	13986482	2.45E-07	0.12	143	–0.44		14.9	TTWH1
qGW1-6^{Farm}	AX-280898927	1	2483022	2.66E-08	0.06	143	–0.66		9.4	TTWH1
qGW1-3^{BF}	AX-115791785	1	43103625	5.18E-10	0.30	143	–0.44		8.7	TTWH1
qGW6-1^{BF}	AX-115737727	6	28484619	5.27E-07	0.15	143	0.48		5.4	Ilpum
qGW12-1^{FSG}	AX-284265976	12	13013702	6.88E-08	0.10	143	–0.39		4.2	TTWH1
qGW8-1^{FSG}	AX-115796459	8	3875546	2.89E-08	0.21	143	0.40		3.7	Ilpum
qGW2-3^{Farm}	AX-279994820	2	35461009	2.02E-06	0.15	143	–0.68		3.5	TTWH1
qGW2-4^{Farm}	AX-154042022	2	24704256	1.30E-09	0.27	143	0.54		3.4	Ilpum
qGW3-3^{Farm}	AX-154797543	3	2973374	4.00E-07	0.31	143	0.39		1.2	Ilpum
	SUPER									
qGW1-1^{BFSG}	AX-273945773	1	5623288	3.39E-09	0.32	143	0.57		15.5	Ilpum
qGW6-2^{FSG}	AX-273990782	6	13986482	2.45E-07	0.12	143	–0.44		14.9	TTWH1
qGW12-1^{FSG}	AX-284265976	12	13013702	6.88E-08	0.09	143	–0.39		4.2	TTWH1
qGW8-1^{FSG}	AX-115796459	8	3875546	2.89E-08	0.21	143	0.40		3.7	Ilpum
	GLM									
qGW1-1^{BFSG}	AX-273945773	1	5623288	3.39E-09	0.32	143	0.57		15.5	Ilpum
qGW6-2^{FSG}	AX-273990782	6	13986482	2.45E-07	0.12	143	–0.44		14.9	TTWH1
qGW12-1^{FSG}	AX-284265976	12	13013702	6.88E-08	0.09	143	–0.39		4.2	TTWH1
qGW8-1^{FSG}	AX-115796459	8	3875546	2.89E-08	0.21	143	0.40		3.7	Ilpum
GT	BLINK									
qGT1^{Blink}	AX-279261704	1	18023142	3.10E-07	0.02	143	–0.07		74.9	TTWH1
	FarmCPU									
qGT2-1^{Farm}	AX-154787777	2	2118477	3.09E-11	0.03	143	–0.13		54.9	TTWH1
qGT2-2^{Farm}	AX-154913392	2	25105471	7.77E-09	0.29	143	0.03	5.3		Ilpum
LWR	BLINK									
qLWR10^{Blink}	AX-115835839	10	22038978	5.11E-07	0.17	143	0.03		26.5	Ilpum
qLWR2-1^{BFSG}	AX-274833045	2	10000097	1.24E-07	0.06	143	–0.09		15.2	TTWH1
qLWR1-1^{BF}	AX-154960834	1	1595394	1.51E-12	0.41	143	–0.04		13.5	TTWH1
qLWR1-2^{Blink}	AX-115737888	1	600441	6.38E-09	0.15	143	–0.06		10.7	TTWH1
qLWR3^{Blink}	AX-154834762	3	8098398	6.51E-10	0.17	143	–0.08		6.9	TTWH1
	FarmCPU									
qLWR2-1^{BFSG}	AX-274833045	2	10000097	1.38E-08	0.06	143	–0.11		32.9	TTWH1
qLWR6-1^{FSG}	AX-115851421	6	10178858	1.54E-16	0.08	143	0.07		30.5	Ilpum
qLWR1-1^{BF}	AX-154960834	1	1595394	9.97E-11	0.41	143	–0.04		13.2	TTWH1
qLWR6-2^{FSG}	AX-155522120	6	30842264	1.26E-06	0.15	143	–0.05		9.4	TTWH1
qLWR8^{FSG}	AX-154176130	8	5398451	4.47E-07	0.15	143	–0.04		5.9	TTWH1
	SUPER									
qLWR2-1^{BFSG}	AX-274833045	2	10000097	1.24E-07	0.06	143	–0.09		15.2	TTWH1
qLWR6-1^{FSG}	AX-115851421	6	10178858	1.54E-16	0.08	143	0.07		30.5	Ilpum
qLWR6-2^{FSG}	AX-155522120	6	30842264	1.26E-06	0.15	143	–0.05		9.4	TTWH1
qLWR8^{FSG}	AX-154176130	8	5398451	4.47E-07	0.15	143	–0.04		5.9	TTWH1
	GLM									
qLWR2-1^{BFSG}	AX-274833045	2	10000097	1.24E-07	0.06	143	–0.09		15.2	TTWH1
qLWR6-1^{FSG}	AX-115851421	6	10178858	1.54E-16	0.08	143	0.07		30.5	Ilpum
qLWR6-2^{FSG}	AX-155522120	6	30842264	1.26E-06	0.15	143	–0.05		9.3	TTWH1

qLWR8^{FSG}	AX-154176130	8	5398451	4.47E-07	0.15	143	–0.04	5.9	TTWH1
TGW	BLINK								
qTGW6^{BFSG}	AX-115737727	6	28484619	3.60E-10	0.15	143	0.88	32.8	Ilpum
qTGW2-1^{BF}	AX-279699609	2	10805604	4.19E-21	0.10	143	–1.33	18. 6	TTWH1
qTGW1-1^{Blink}	AX-154298059	1	5644298	2.56E-06	0.30	143	0.37	11.6	Ilpum
qTGW3-1^{BF}	AX-154471576	3	15332432	1.08E-10	0.06	143	–0.93	10.7	TTWH1
qTGW2-2^{BF}	AX-154096541	2	10773042	1.05E-11	0.17	143	0.83	7.8	Ilpum
qTGW1-2^{BFSG}	AX-154333920	1	5860250	1.95E-10	0.30	143	0.40	4.9	Ilpum
qTGW1-3^{BF}	AX-154810092	1	42931550	2.15E-17	0.26	143	–0.62	4.03	TTWH1
	FarmCPU								
qTGW6^{BFSG}	AX-115737727	6	28484619	9.95E-08	0.15	143	0.71	54.0	Ilpum
qTGW2-1^{BF}	AX-279699609	2	10805604	5.49E-15	0.10	143	–0.98	27.9	TTWH1
qTGW3-2^{Farm}	AX-123153600	3	7887961	2.47E-19	0.33	143	0.84	13.9	Ilpum
qTGW1-3^{BF}	AX-154810092	1	42931550	4.87E-17	0.26	143	–0.58	8.3	TTWH1
qTGW2-2^{BF}	AX-154096541	2	10773042	3.38E-10	0.17	143	0.72	7.5	Ilpum
qTGW3-1^{BF}	AX-154471576	3	15332432	4.98E-09	0.06	143	–0.79	7.2	TTWH1
qTGW1-2^{BFSG}	AX-154333920	1	5860250	1.41E-11	0.30	143	0.57	6.2	Ilpum
	SUPER								
qTGW6^{BFSG}	AX-115737727	6	28484619	9.95E-08	0.15	143	0.71	54.0	Ilpum
qTGW1-2^{BFSG}	AX-154333920	1	5860250	1.41E-11	0.30	143	0.57	6.2	Ilpum
	GLM								
qTGW6^{BFSG}	AX-115737727	6	28484619	9.95E-08	0.15	143	0.71	54.0	Ilpum
qTGW1-2^{BFSG}	AX-154333920	1	5860250	1.41E-11	0.30	143	0.57	6.2	Ilpum

GL: grain length, GW: grain width, GT: grain thickness, LWR: Length-to-width ratio, and TGW: thousand-grain weight. Chr: chromosome, MAF: minor allelic frequency, nobs: number of observations, PVE: phenotype variance explained. *qTrait*^{Blink}: QTL detected by BLINK only, *qTrait*^{Farm}: QTL detected by FarmCPU only, GLM, *qTrait*^{Glm}: QTL detected by GLM only, *qTrait*^{FSG}: QTL co-detected by FarmCPU, SUPER, and GLM, *qTrait*^{BFSG}: QTL co-detected by BLINK, FarmCPU, SUPER, and GLM.

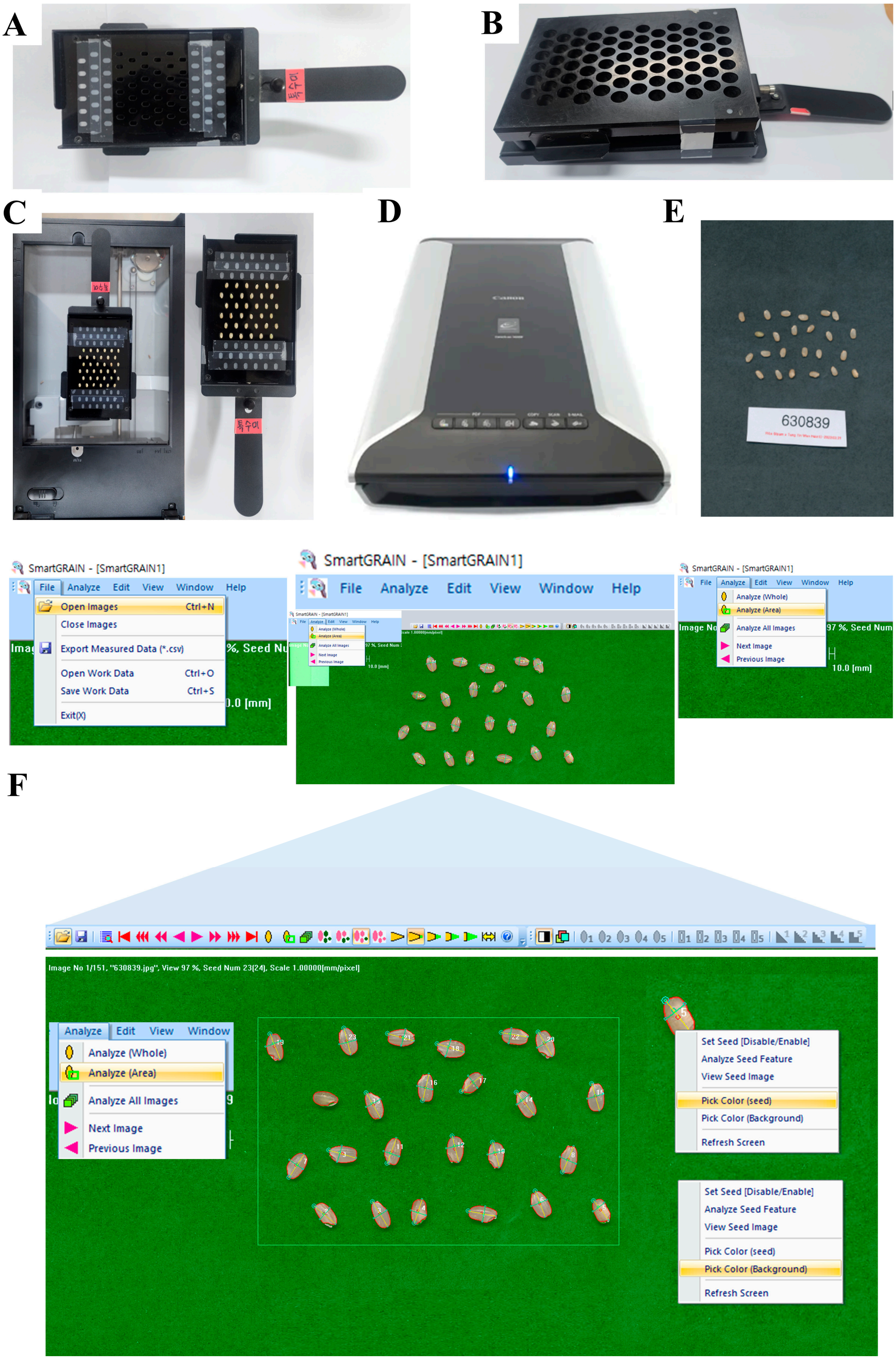

Figure S1. Illustration of rice grain length, width, and length-to-width ratio analysis using SMART GRAIN software. (**A**,**B**) Images of a rice grain dispenser, (**C**) A rice dispenser with rice grain on a scanner, (**D**,**E**) Scanner and scanned rice grains with a label, and (**F**) visualization of stepwise grain shape analysis.
